# Morphological and ontogenetic characteristics of *Miridex putorii* (Acariformes: Demodecidae), a new genus and species of skin mite specific to the European polecat *Mustelaputorius*

**DOI:** 10.1016/j.ijppaw.2022.06.005

**Published:** 2022-06-22

**Authors:** Joanna N. Izdebska, Leszek Rolbiecki, Steffen Rehbein

**Affiliations:** a Department of Invertebrate Zoology and Parasitology, Faculty of Biology, University of Gdańsk, Wita Stwosza 59, 80-308, Gdańsk, Poland; b Boehringer Ingelheim Vetmedica GmbH, Kathrinenhof Research Center, Walchenseestr. 8-12, 83101, Rohrdorf, Germany

**Keywords:** New genus, Acariformes, Demodecid mites, Carnivorans, Mustelidae

## Abstract

Among carnivorans, mites of the family Demodecidae are mainly represented by the eight species of the genus *Demodex* known to cause *demodecosis* in domestic dog *Canis lupus familiaris* Linnaeus, 1758 and domestic cat *Felis catus* Linnaeus, 1758. However, nine other *Demodex* species from wild carnivorans are also known; in addition they are only known from few records. Previously unknown demodecid mites have been isolated from European polecats, *Mustela putorius* Linnaeus, 1758, originating from Germany. The specimens are characterized of by an aedeagus with a posterior end located between the opisthosoma and podosoma and an anterior end in the gnathosoma area, with a genital opening in the epistome area; aedeagus length corresponds to 53% (45–59%) of male body length. The mites were isolated from the head skin in 16 of 21 polecats examined (76.2%), mainly in the mystacial vibrissae area (84.8%) and less often in adjacent areas. However, the mite infestation did not appear to cause skin abnormality. Based on the morphological analysis of the adult mites and their morphological ontogenesis, including significant characteristics in demodecid taxonomy, the mite specimens have been classified as representatives of a new species and genus, described as *Miridex putorii* gen. nov., sp. nov.

## Highlights

• A new genus for the family Demodecidae.
• A new species and first record from demodecid mites in the European polecat *Mustela putorius*.
• Peculiar features of males: genital opening located on the gnathosoma and aedeagus constitutes above 50% of body length.

## Graphical abstract

**Figure:**
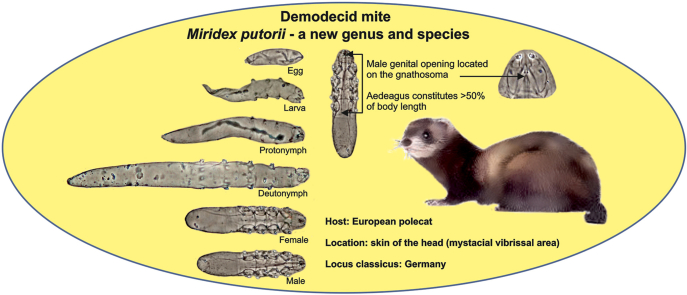

## 1 Introduction

The Demodecidae (Acariformes: Prostigmata) of carnivoran mammals have been relatively poorly studied. Most information regarding mites of the genus *Demodex* concern domestic animals, dogs and cats, each harboring four species; however, some of these have been discovered only in recent years (Izdebska and Rolbiecki, 2018, 2020; Morita et al., 2018). The majority of known information concerns the clinical signs of the parasitosis in the hosts, *canine demodecosis* and *feline demodecosis*, respectively.

In other carnivorans, particularly wild species, demodecid mites are only reported from isolated publications. Thus far, two species have been found in bears (Xu et al., 1986; Desch, 1995), two in large felids (Shi et al., 1985; Desch, 1993) and two in pinnipeds (Dailey and Nutting, 1979; Desch et al., 2003; Izdebska and Rolbiecki, 2020; Izdebska et al., 2020). In addition, only three demodecid mite species have been described among the species-rich mustelid group: *Demodex melesinus* Hirst, 1921 from the European badger *Meles meles* (Linnaeus, 1758) described from the United Kingdom and Poland (Hirst, 1921; Izdebska et al., 2018), *D. lutrae* Izdebska et Rolbiecki, 2014 from the Eurasian otter *Lutra lutra* (Linnaeus, 1758) (Izdebska and Rolbiecki, 2014; Rolbiecki and Izdebska, 2014), described from Poland, and *D. erminae* Hirst, 1919 from the ermine *Mustela erminea* Linnaeus, 1758, found in the United Kingdom and New Zealand (Hirst, 1919; Nutting et al., 1975).

The present study describes the discovery of Demodecidae mites from the European polecat *M. putorius* Linnaeus, 1758 originating from Germany. The new taxon exhibits characteristics that are distinct not only from all known demodecid mite species, but also from the eight genera known thus far; therefore, it has been classified in a separate, new genus as *Miridex putorii* gen nov., sp. nov.

## 2 Materials and methods

In the course of a survey of the parasite fauna of the European polecat in Germany (Kretschmar, 2016), the skin of the head of 21 animals collected during the period of October 2013 to August 2015 was examined for demodecid mites. The 21 polecats originated from the Federal States Lower Saxony (county Aurich, 53°28′15″N, 07°28′59″E: six specimens), Northrhine Westfalia (county Bonn, 50°44′00″ N, 07°06′00″E: one specimen; county Borken, 52°02′07″ N, 06°49′28″E: five specimens; county Heinsberg, 51°06′00″ N, 06°09′00″E: five specimens), Hessia (county Hersfeld-Rotenburg, 50°53′14″ N, 10°00′20″E: one specimen; county Wetteraukreis, 50°26′06″ N, 08°40′08″E: one specimen) and Bavaria (county Nürnberger Land, 50°45′31″ N, 12°42′36″E: two specimens).

The demodecid mites were recovered by digestion of host skin fragments (Izdebska, 2004). The method was modified to suit the examined host. Skin fragments of 1 cm^2^ were taken from several head regions, including the area around the eyes, nose, vibrissae, lips, chin, cheeks, and vertex. The samples, which had been preserved in 70% ethanol, were subjected to digestion in 10% potassium hydroxide solution. The resulting solution was decanted and examined under phase-contrast microscopy (Nikon Eclipse 50i); a 1 cm^2^ of skin sample yielded approximately 100 wet preparations. Any mites were placed in polyvinyl-lactophenol solution, measured (micrometers), photographed, and graphically documented. The following measurements were made: total body length = length of gnathosoma, podosoma and opisthosoma; gnathosomal width = width at base; podosomal and opisthosomal width = maximum width.

The specimen depository are cited using the abbreviation UGDIZP, University of Gdańsk, Department of Invertebrate Zoology and Parasitology, Gdańsk, Poland (Zhang, 2018).

The species description adopted the nomenclature commonly used for the Demodecidae (Nutting, 1976). It was completed with the nomenclature proposed by Bochkov (2008) for the superfamily Cheyletoidea (Acariformes: Prostigmata) and by Izdebska and Rolbiecki (2016). The scientific and common names of the hosts follow Wilson and Reeder (2005) and the Integrated Taxonomic Information System (2022).

## 3 Results

### 3.1 Description

#### 3.1.1 *Miridex* gen. nov.

**Fig. 1:**
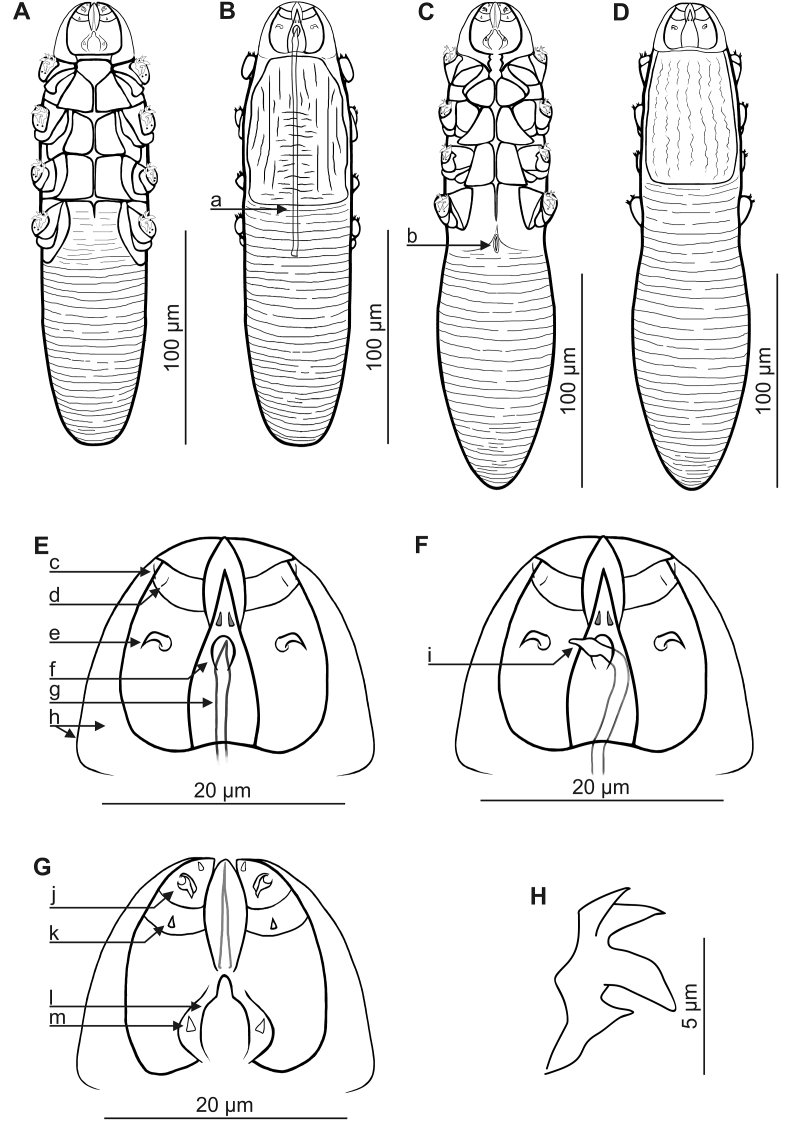
*Miridex putorii* gen. nov., sp. nov. A, male, ventral view; B, male, dorsal view, a. aedeagus; C, female, ventral view, b. vulva; D, female, dorsal view; E, gnathosoma, male, dorsal view, c. seta *dG*, d. seta *dF*, e. supracoxal spine (seta *elc.p*), f. genital opening, g. anterior end of aedeagus, h. membrane capitulum; F, gnathosoma, male, dorsal view, i. everted anterior part of aedeagus; G, gnathosoma, male, ventral view, j. spines on palps, k. seta *v”F*, l. pharyngeal bulb, m. subgnathosomal seta (seta *n*); H, claw on the leg.

**Fig. 2:**
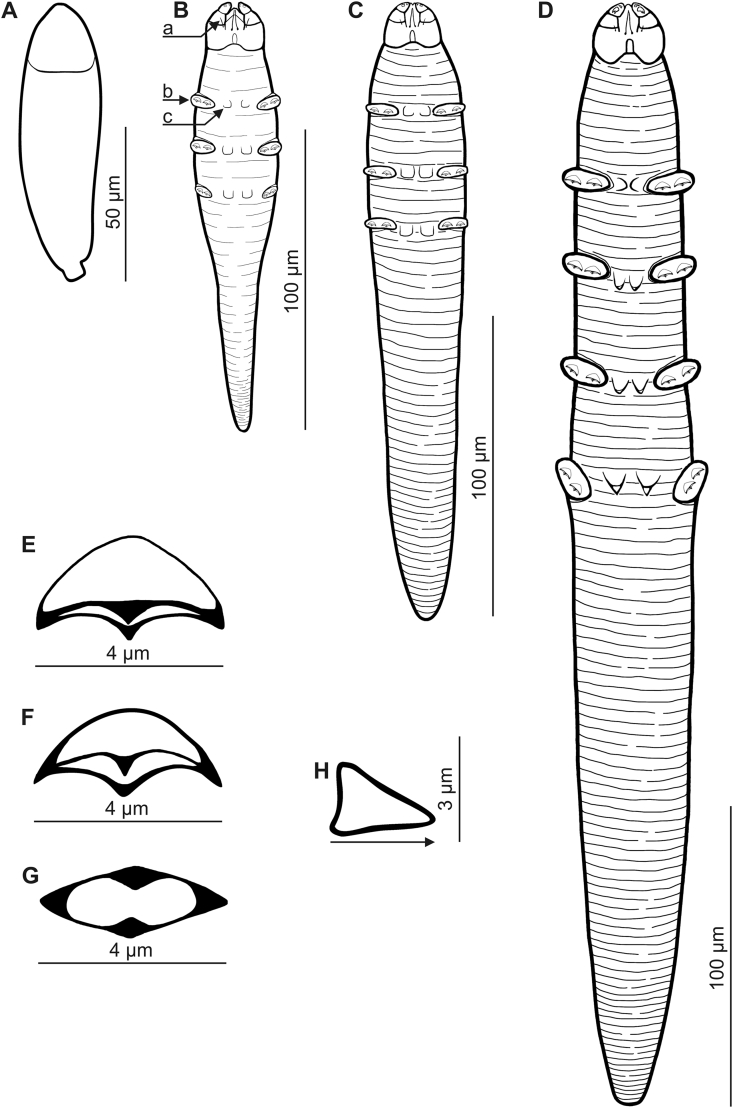
*Miridex putorii* gen. nov., sp. nov., egg and immature stages. A, egg; B, larva, ventral view, a. vimineous seta, b. leg with claws, c. ventral scutum; C, protonymph, ventral view; D, deutonymph, ventral view; E, F, G, claw, various views; H, supracoxal spine of deutonymph, arrow indicate the orientation of left spine as viewed on the gnathosoma.

The genus *Miridex* displays all features of the Demodecidae, indicated by Baker and Wharton (1952) and amended by Desch et al. (1972), Bukva (1996) and Izdebska and Rolbiecki (2016). It is characterized by the presence of a male genital opening on the epistome of gnathosoma. Furthermore, gnathosoma is clearly separated, with well-developed membrane capitulum; pedipalps with relatively long, clearly separated segments. Legs with five well-developed, clearly separate free segments and two massive claws on each tarsus, strongly bifurcated at the end. In larvae, one pair of long, vimineous, and well-developed setae present at inner edge of anterior margin of basal segment of pedipalps (Fig. 1, Fig. 2).

### 3.2 Descriptions

#### 3.2.1 *Miridex putorii* sp. nov.

**Fig. 3:**
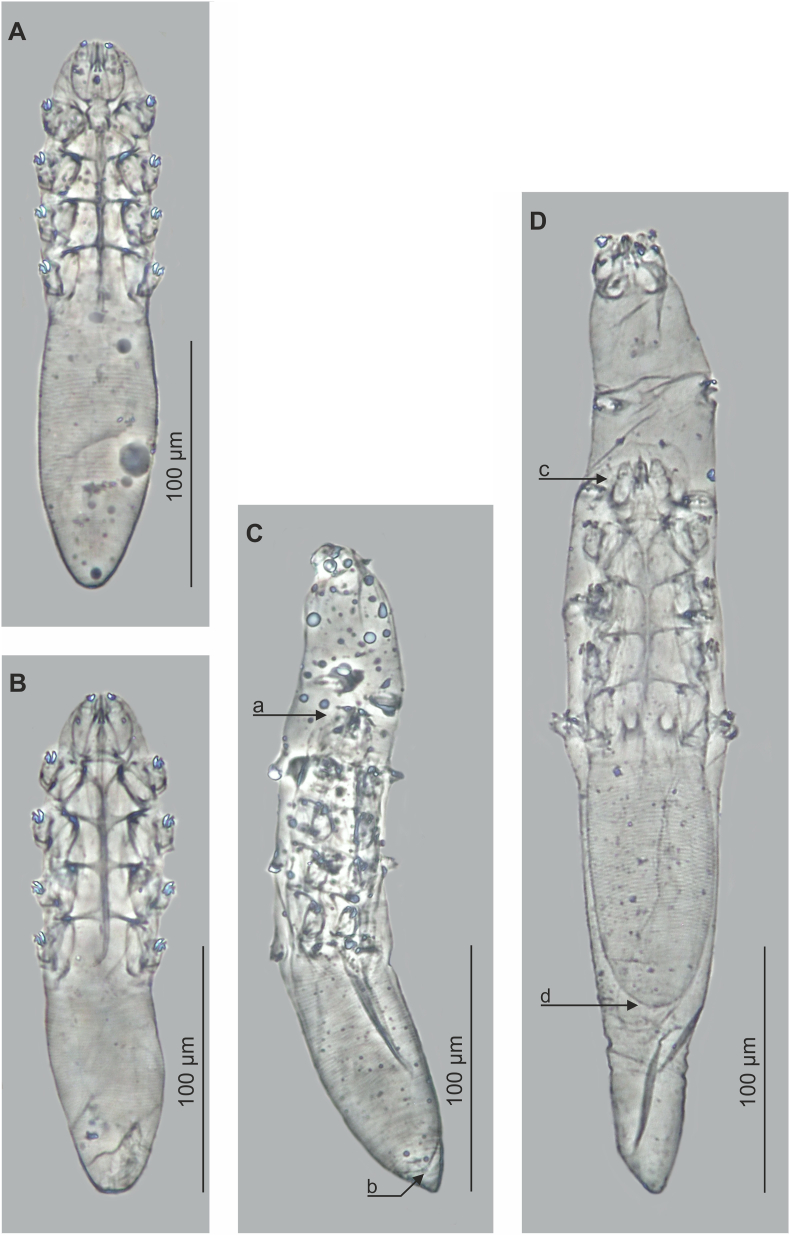
*Miridex putorii* gen. nov., sp. nov. A, female, ventral view; B, male, ventral view; C, pharate male, deutonymph with visible male inside, a. anterior end of male gnathosoma, b. posterior end of male opisthosoma; D, pharate female, deutonymph with visible female inside, c. anterior end of female gnathosoma; d. posterior end of female opisthosoma.

MALE (n = 1 holotype and 55 paratypes; Fig. 1, Fig. 3, Table 1): Body, cylindrical, 207 (186–223) long and 50 (43–56) wide (holotype 208 x 47). Gnathosoma oval (length close to width at base); surrounded by wide membranae capitulum. Palps consist of three clearly-separated segments; basal (coxal) segment wide, next two (trochanter–femur–genu, tibia–tarsus) elongated, narrow at anterior end. On dorsal surface of basal segments at external edges, hooked supracoxal spines (setae *elc.p*) present, ca. 2 long (holotype, 2); each palp terminating in three spines: two large spines (one bifurcated), ca. 3 long (holotype, 3) and one small, conical, ca. 1.5–2 long (holotype, 2); also relatively strongly-developed setae *dG* and *dF*, and large, conical setae *v”F* on middle segment (trochanter–femur–genu) present. On ventral part of gnathosoma, funnel-shaped pharyngeal bulb, with pair of relatively large, conical subgnathosomal setae (setae *n*) located half-way, or below, along pharyngeal bulb on both sides. Podosoma cylindrical; on dorsal side podosomal shield present, reaching level of legs III. Four pairs of short legs, with coxa integrated into ventral idiosomal wall and clearly separated five free segments; coxa with large, triangular spines, located medially; two strongly bifurcated, massive claws, ca. 7 long (holotype, 7) with large, hooked spur on each tarsus; additionally, two large, conical spines present in anterior part of tibia. Epimeral plates (coxal fields) trapezoidal, distinctly sclerotized; I–III epimeral plates connect medially; pair IV poorly separated, only anterior edge clearly visible. Opisthosoma constitutes 45% (40–51%) of body length (holotype, 42%); cylindrical, rounded at end; width slightly smaller or close to podosoma. Whole opisthosoma clearly, densely annulated; annuli ca. 1 wide (holotype, 1); annulations reach posterior edge of podosomal shield on dorsal side of podosoma and IV epimeral plates on ventral side of podosoma. Opisthosomal organ absent. Aedeagus 109 (100–123) long (holotype, 107), constitutes 53% (45–59%) of body length (holotype, 51%), narrow, bar-shaped, on dorsal side, located from border of opisthosoma and podosoma to middle part of gnathosoma; genital opening located on dorsal side, at anterior part of epistome.

**Table 1:** Body size for adults of *Miridex putorii* gen. nov.

Morphologic features	Male (n = 56) Mean ± SD (range)	Female (n = 51) Mean ± SD (range)
Length of gnathosoma	24 ± 1 (23–26)	25 ± 1 (23–27)
Width of membrane capitulum	32 ± 3 (26–40)	32 ± 3 (26–38)
Width of gnathosoma (at base)	25 ± 1 (23–27)	25 ± 1 (23–27)
Length of podosoma	89 ± 4 (80–100)	90 ± 4 (82–100)
Width of podosoma	50 ± 3 (43–56)	49 ± 2 (45–56)
Length of opisthosoma	93 ± 7 (78–113)	113 ± 9 (95–130)
Width of opisthosoma	48 ± 3 (38–55)	49 ± 2 (45–57)
Aedeagus	109 ± 5 (100–123)	–
Vulva	–	10 ± 1 (8–12)
Total length of body	207 ± 8 (186–223)	227 ± 11 (201–246)
Body length to width ratio	4.2:1 ± 0.3:1 (3.7:1–4.9:1)	4.6:1 ± 0.3 (3.9:1–5.4:1)
Opisthosoma length to body length ratio (%)	45 ± 2 (40–51)	50 ± 2 (45–54)
Gnathosoma length to width ratio	1.0:1 ± 0.03:1 (0.9:1–1.0:1)	1.0:1 ± 0.03:1 (0.9:1–1.0:1)
Aedeagus length to body length ratio (%)	53 ± 3 (45–59)	–

FEMALE (n = 51 paratypes; Fig. 1, Fig. 3, Table 1): Usually slightly longer than males: 227 (201–246) long and 49 (45–57) wide. Gnathosoma of similar shape and morphological details to males. Podosoma cylindrical, wide; on dorsal side of podosoma podosomal shield present, reaching level of legs III. Legs clearly separated, similar to those in males. Epimeral plates trapezoidal, distinctly sclerotized; I–III epimeral plates connect medially; pair IV poorly separated, only anterior edge clearly visible; posterior edges of pair IV forming triangular incision. Opisthosoma usually longer than males, constitutes 50% (45–54%) of body length; width close or greater than podosoma; cylindrical, wide, rounded at end, and very delicate but densely annulated. Opisthosomal organ absent. Vulva 10 (8–12) long; tubular, located below posterior edge of epimeral plates IV.

EGG (n = 11 eggs; Fig. 2): Operculate, club-shaped, 93 (88–100) long and 26 (23–28) wide.

LARVA (n = 3 paratypes; Fig. 2, Table 2): Club-shaped, stocky, 143 (132–152) long and 26 (25–28) wide; length-to-width ratio 5.4:1 (5.3:1–5.6:1). Gnathosoma relatively large, distinctly separated, trapezoidal. Palps 3-segmented; all segments clearly separated, narrow, elongated, the basal segment large. On dorsal surface, in central part of basal segments, wedge-shaped supracoxal spines (setae *elc.p*) present, ca. 2–3 long. Terminal segments of palps topped with two spines (one claw-like and one fine spine). On ventral surface of gnathosoma, horseshoe-shaped pharyngeal bulb; at inner edge of anterior margin of basal segment, pair of long setae present, ca. 3 long, probably corresponding to subgnathosomal setae. Three pairs of unsegmented, clearly separated legs; pairs I and II larger, ca. 13 long than pair III ca. 9–10 long; each legs equipped with two 4-pointed claws, ca. 1.5 (for III legs) wide and ca. 2 (for I and II legs) wide; also three pairs of oval ventral scutes, located between I–III pairs of legs present. Opisthosoma conical, constitutes 54% (49–57%) of body length. Podosoma and opisthosoma densely annulated.

**Table 2:** Body size for immature stages of *Miridex putorii* gen. nov.

Morphologic features	Larva (n = 3) Mean ± SD (range)	Protonymph (n = 6) Mean ± SD (range)	Deutonymph (n = 42) Mean ± SD (range)
Length of gnathosoma	15 ± 0 (15–15)	18 ± 2 (15–20)	22 ± 2 (16–25)
Width of gnathosoma	20 ± 2 (18–22)	20 ± 2 (18–22)	24 ± 3 (16–30)
Length of podosoma	51 ± 10 (42–62)	58 ± 5 (53–68)	148 ± 28 (55–183)
Width of podosoma	26 ± 2 (25–28)	32 ± 6 (25–40)	46 ± 8 (35–65)
Length of opisthosoma	77 ± 3 (75–80)	126 ± 21 (105–160)	194 ± 36 (123–259)
Width of opisthosoma	25 ± 1 (24–25)	30 ± 6 (25–40)	41 ± 7 (29–58)
Total length of body	143 ± 10 (132–152)	203 ± 25 (173–246)	364 ± 52 (236–449)
Body length to width ratio	5.4:1 ± 0.1:1 (5.3:1–5.6:1)	4.0:1 ± 1.0:1 (3.0:1–5.7:1)	8.0:1 ± 1.4:1 (5.2:1–11.0:1)
Opisthosoma length to body length ratio (%)	54 ± 4 (49–57)	62 ± 3 (57–65)	53 ± 6 (38–69)
Gnathosoma length to width ratio	0.8:1 ± 0.1:1 (0.7:1–0.8:1)	0.9:1 ± 0.1:1 (0.7:1–1.0:1)	0.9:1 ± 0.1:1 (0.7:1–1.1:1)

PROTONYMPH (n = 6 paratypes; Fig. 2, Table 2): Protonymph similar to larva but longer and slender, and more cylindrical, 203 (173–246) long and 32 (25–40) wide, length-to-width ratio 4.0:1 (3.0:1–5.7:1). Shape of gnathosoma and morphological details similar to those in larvae, but subgnathosomal setae shorter than in larvae. Three pairs of unsegmented, clearly separated, same-size legs; each leg equipped with two 4-pointed claws, ca. 3 wide; also three pairs of oval ventral scutes topped with triangular spurs, located between I–III pairs of legs present. Opisthosoma cylindrical, constitutes 62% (57–65%) of body length. Podosoma and opisthosoma distinctly, densely annulated.

DEUTONYMPH (n = 42 paratypes; Fig. 2, Fig. 3, Table 2): Cylindrical, slightly spindle-shaped (widest in middle of podosoma). Deutonymph larger, more elongated than protonymph, 364 (236–449) long and 46 (35–65) wide; length-to-width ratio 8.0:1 (5.2:1–11.0:1). Shape of gnathosoma and morphological details similar to those in protonymphs, but supracoxal spines longer (ca. 3–4) than in protonymphs. Four pairs of unsegmented, clearly separated, same-size legs; each leg equipped with two 4-pointed claws, ca. 4 wide; also four pairs of oval ventral scutes topped with triangular spurs, located between I–IV pairs of legs present. Opisthosoma, cylindrical, constitutes 53% (38–69%) of body length. Podosoma and opisthosoma distinctly, densely annulated.

In development, adult stages are located in the middle region of deutonymph (gnathosoma of adults at level of I–II pair of deutonymph legs); furthermore, two deutonymph morphotypes were observed, one with a shorter (Fig. 3C) and one with a longer (Fig. 3D) opisthosoma: the former probably male and latter female.

### 3.3 ZooBank registration

The Life Science Identifier for *Miridex* gen. nov. is urn:lsid:zoobank.org:act:A3EAB7E5-9892-408C-A84C-D8E351FD9131 and for *Miridex putorii* sp. nov. is urn:lsid:zoobank.org:act:1C89C9BC-D77C–4C53-AB69-71821201392F.

### 3.4 Material deposition

Holotype male (reg. no. UGDIZPMMpDMp13m); mystacial vibrissal; host *Mustela putorius* (reg. nos. MCMMp116/2014); county Heinsberg, Germany; November 2014; parasite coll. J.N. Izdebska; host coll. S. Rehbein; the whole-type material (mounted microscope slide with the demodecid mite) deposited in the scientific collections within the framework of the Collection of Extant Invertebrates in the Department of Invertebrate Zoology and Parasitology, University of Gdańsk, Poland (UGDIZP). Paratypes 55 males (reg. no. UGDIZPMMpDMp01m−12m, UGDIZPMMpDMp14m−56), 51 females (reg. no. UGDIZPMMpDMp01f−51f), three larvae (reg. no. UGDIZPMMpDMp01l−03l), six protonymphs (reg. no. UGDIZPMMpDMp01pn−06pn), 42 deutonymphs (reg. no. UGDIZPMMpDMp01dn−42dn); mainly mystacial vibrissal area, less often in adjacent areas; host *Mustela putorius* (reg. nos. MCMMp75/2014, MCMMp76/2014, MCMMp79/2014, MCMMp82/2014, MCMMp85/2015, MCMMp87/2015, MCMMp89/2013, MCMMp90/2014, MCMMp92/2014, MCMMp94/2013, MCMMp96/2014, MCMMp98/2014, MCMMp105/2014, MCMMp111/2014, MCMMp116/2014, MCMMp118/2015); counties Aurich, Borken, Heinsberg, Wetteraukreis, Nürnberger Land, Germany; October 2013, February 2014, August 2014, October 2014, November 2014, December 2014, January 2015, February 2015; parasites coll. J.N. Izdebska and L. Rolbiecki; host coll. S. Rehbein; the whole-type material (mounted microscope slides with the demodecid mites) deposited in the scientific collections within the framework of the Collection of Extant Invertebrates in the Department of Invertebrate Zoology and Parasitology, University of Gdańsk, Poland (UGDIZP).

### 3.5 Etymology

As is common practice regarding the creation of generic names in the Demodecidae, a name was set combining *mirus* (peculiar, unusual), referring to the peculiar feature (regarding location of the male genital opening and the length aedeagus) with the word *dex* – ‘a worm’. In turn, the specific epithet *putorii* was added to refer to the host species name.

### 3.6 Infestation and location in the host

*Miridex putorii* sp. nov. was found in 16 of 21 European polecats examined (76.2%); in total, 158 specimens (56 males, 51 females, three larvae, six protonymphs, 42 deutonymphs) and 11 eggs were found. Mites were found in the skin of the mystacial vibrissal area (134 specimens, 84.8%) and in adjacent areas (24 specimens, 15.2%); eggs were found only in the mystacial vibrissal area. The infestation did not cause skin lesions in the mite positive polecats.

### 3.7 Differential diagnosis

The feature distinguishing *M. putorii* sp. nov. from other known Demodecidae is the genital opening of the male, which is located on the dorsal side of the gnathosoma (on the epistome); in other genera, it is always located on the dorsal side of the podosoma. Another specific feature is the presence of the massive claws, strongly bifurcated at the end; in *Demodex*, they are clearly less bifurcated, with different shapes being observed in other genera. *Miridex putorii* sp. nov. most closely resembles some *Demodex* species in the habit, shape and arrangement of its epimeral plates; in these species, the epimeral plates come into contact in the midline of the ventral part of the body. However, the palps and legs of *Demodex* are strongly shortened, being composed of overlapping segments which are sometimes difficult to distinguish. While *M. putorii* sp. nov. resembles *Glossicodex*, insofar that its leg segments are clearly separated, in the case of the latter, the leg segments are elongated and end with hooked, non-bifurcated claws. In addition, a relatively large, conical subgnathosomal setae can be found on both sides of the pharyngeal bulb on *M. putorii* sp. nov. These are pronounced in the larvae in the form of long, vimineous setae. In other Demodecidae, the subgnathosomal setae are typically fine, and often difficult to observe.

## 4 Discussion

Our taxonomic analysis, including data from the morphological ontogenesis (including juvenile stages) supports that the specimens identified from the European polecat should be classified into a new (separate) genus. This genus appears to exhibit certain archaic and specific features which reflect unique adaptations and whose significance is difficult to interpret.

The representatives of individual Demodecidae genera can often be distinguished based on the structure of the legs (i.e. the shape of the claws and the shape and arrangement of the epimeral plates), as well as the form and location of the structures related to the gnathosoma (Fain, 1959; Lukoschus and Nutting, 1979; Bukva, 1996; Izdebska and Rolbiecki, 2016). However, it should be added that these features are not homogeneous within the genus *Demodex*, and they should be subject to revision.

In hitherto described Demodecidae species, the female's vulva is located on the ventral side of the body; however, it is observed at different levels, ranging from the border between podosoma and opisthosoma (partially within IV. pair of epimeral plates) or within the opisthosoma, directly under the edge of the last pair of epimeral plates, or even lower. In contrast, the genital opening of the male is always located on the dorsal side of the podosoma. Its precise position depends on the location of the aedeagus (whose length corresponds to approx. 10–15% length of the male body); in *M. putorii* sp. nov. the aedeagus is very long, corresponding to 45–59% of male's body length, and it reaches from the margin of podosoma and opisthosoma to the mid-gnathosoma, with an opening within the epistome. Among the known demodecid mites, a relatively long aedeagus is characteristic of *Glossicodex musculi* Izdebska et Rolbiecki, 2016, however, it covers a space corresponding to three epimeral plates (corresponding to 20–25% of male body length) with a genital opening located on the podosoma (Izdebska and Rolbiecki, 2016). Therefore, the aedeagus location observed for *M. putorii* sp. nov. is a unique feature within the Demodecidae.

On the other hand, the clear separation of palps and leg segments, claw-like spines on the palps, or larger, more distinct setae, particularly in juvenile stages, may be of a primary nature. Most importantly, the long, vimineous subgnathosomal setae on the gnathosoma of juvenile stages form a clear link to other Cheyletoidea, which are phylogenetically more distinct from Demodecidae (Bochkov, 2008).

The skin area inhabited by *M. putorii* sp. nov. is a typical location for various Demodecidae. For instance, *D. gracilentus* Izdebska et Rolbiecki, 2013 exhibits a strong topical specificity, being restricted to the vibrissae region of the striped field mouse *Apodemus agrarius* (Pallas, 1771), while *D. vibrissae* Izdebska, Rolbiecki et Fryderyk, 2016 occupies the vibrissae region of the house mouse *Mus musculu*s Linnaeus, 1758. The skin of the head is also a characteristic location for demodecid mites parasitising the domestic cat *Felis catus* Linnaeus, 1758, e.g. *D. murilegi* Izdebska, Rolbiecki et Fryderyk, 2022 was found solely in the region of the lips, nose and chin, whereas *D. cati* Megnin, 1877 prefers the nasal region, areas of the eyes and auricles (Izdebska and Rolbiecki, 2013; Izdebska et al., 2016, 2022).

The lack of data regarding the Demodecidae in the large, biodiverse and widely distributed mustelid group can probably be attributed to the asymptomatic nature of the infestation. *Demodecosis* is usually known from humans and domestic animals, but constitutes a rare observation in wild animals, even at high infestation levels: no skin abnormalities were observed in the case of *D. melesinus* in the European badger or *D. lutrae* in the Eurasian otter, despite very high mite densities (Izdebska and Rolbiecki, 2014; Rolbiecki and Izdebska, 2014; Izdebska et al., 2018).

More surprising is, however, the scarcity of data regarding demodecid mites from mustelids farmed for fur or kept as pets, such as the species of the genus *Mustela*. While a report of “*Demodex bonapartei*” Nutting, 1950 from *M. erminea cicognanii* Bonaparte, 1838 has been given in international literature (Nutting, 1950, 1964), no species description has yet been published: the report is included in an unpublished doctoral thesis. A report also exists on *demodecosis* associated with local skin lesions (alopecia, pruritus) in ferrets *M. putorius furo* Linnaeus, 1758, caused by the adult and juvenile forms of unidentified *Demodex* (Noli et al., 1996). These mites were found in skin scrapings and exudate from the ear of two ferrets; they were very small, with short, blunt opisthosoma and were reminiscent of *D. criceti* Nutting et Rauch, 1958 from *Mesocricetus auratus* (Waterhouse, 1839) or to the unnamed cat mite (Noli et al., 1996). Furthermore, while information exists on the isolation of an unknown *Demodex* from the long-tailed weasel *M. frenata* Lichtenstein, 1831 and *M. putorius putorius* Linnaeus, 1758, no detailed data have been provided (Noli et al., 1996).

Many mammals have been found to demonstrate synhospital, co-occurring Demodecidae species, these being specific for the given host species, and occupying different locations (Izdebska and Rolbiecki 2020). It is therefore likely that further representatives of this mite family will be found in the European polecat and other members of the Mustelidae.

## Declaration of competing interest

Authors have no conflict of interest to declare.
